# Elucidation of the molecular responses of a cucumber segment substitution line carrying *Pm5.1* and its recurrent parent triggered by powdery mildew by comparative transcriptome profiling

**DOI:** 10.1186/s12864-016-3438-z

**Published:** 2017-01-05

**Authors:** Qiang Xu, Xuewen Xu, Yang Shi, Xiaohua Qi, Xuehao Chen

**Affiliations:** Department of horticulture, School of horticulture and plant protection, Yangzhou University, 48 east wenhui road, Yangzhou, Jiangsu 225009 China

**Keywords:** Cucumber, Powdery mildew, Segment substitution, Resequencing, RNA-sequencing

## Abstract

**Background:**

Powdery mildew (PM) is one of the most severe fungal diseases of cucurbits, but the molecular mechanisms underlying PM resistance in cucumber remain elusive. In this study, we developed a PM resistant segment substitution line SSL508-28 that carried a segment on chromosome five representing the *Pm5.1* locus from PM resistant donor Jin5-508 using marker-assisted backcrossing of an elite PM susceptible cucumber inbred line D8.

**Results:**

Whole-genome resequencing of SSL508-28, Jin5-508 and D8 was performed to identify the exact boundaries of the breakpoints for this introgression because of the low density of available single sequence repeat markers. This led to the identification of a ~6.8 Mb substituted segment predicted to contain 856 genes. RNA-seq was used to study gene expression differences in PM treated (plants harvested 48 h after inoculation) and untreated (control) SSL508-28 and D8 lines. Exactly 1,248 and 1,325 differentially expressed genes (DEGs) were identified in SSL508-28 and D8, respectively. Of those, 88 DEGs were located in the ~6.8 Mb segment interval. Based on expression data and annotation, we identified 8 potential candidate genes that may participate in PM resistance afforded by *Pm5.1*, including two tandemly arrayed genes encoding receptor protein kinases, two transcription factors, two genes encoding remorin proteins, one gene encoding a P-type ATPase and one gene encoding a 70 kDa heat shock protein. The transcriptome data also revealed a complex regulatory network for *Pm5.1*-mediated PM resistance that may involve multiple signal regulators and transducers, cell wall modifications and the salicylic acid signaling pathway.

**Conclusion:**

These findings shed light on the cucumber PM defense mechanisms mediated by *Pm5.1* and provided valuable information for the fine mapping of *Pm5.1* and breeding of cucumber with enhanced resistance to PM.

**Electronic supplementary material:**

The online version of this article (doi:10.1186/s12864-016-3438-z) contains supplementary material, which is available to authorized users.

## Background

Cucumber is an important vegetable crop and is widely cultivated in the world with total harvest of more than two million hectares in 2016, ranking 4^th^ in quantity of world vegetable production (FAO STAT 2016, http://faostat3.fao.org). Powdery mildew (PM hereinafter), mainly caused by *Podosphaera fusca* [1, 2], is among the most destructive fungal diseases amongst Cucurbitaceae family crops including cucumber, melon, watermelon, pumpkin and squash [3–5]. Because cucumbers are grown year-round in greenhouses or high tunnels, PM finds such sheltered conditions favorable, has had a profound impact on the cucumber industry [6]. The typical symptoms of the disease are decline in leaf photosynthetic capacity and reduction in fruit quality [7]. In most cucumber production areas, fungicide application is the major method of PM disease control [8]. In the European Union, over 80,000 tons of fungicides are applied annually to control PM, which corresponds to about 67% of the total fungicides used for all crops [9]. However, excessive fungicide use will not only increase selection pressure on PM pathogen populations to adapt increasing levels of fungicide resistance, but it also detrimental to human health and the environment [10]. Thus, development of cucumber cultivars with enhanced tolerance to PM is a favored strategy for PM disease control.

A prerequisite for crop improvement is a better understanding of the molecular defense mechanisms involved in disease resistance. Quantitative trait loci (QTLs) associated with PM resistance have been mapped to six cucumber chromosomes [1, 2, 8, 11–13]. Although these findings have provided insights into the genetic control of cucumber PM resistance, the molecular defense mechanisms of host resistance against cucumber PM remain elusive. Genetic and physiological plasticity allow plants to adapt to a changing environment, but such plasticity requires sophisticated regulatory mechanisms to simultaneously alter the expression of various genes [14]. Comparative transcriptomic analysis is, therefore, necessary for the interpretation of the functional elements of the host genome and extending our understanding of disease development [10]. Transcriptome profiling of immune responses in plants, such as *Arabidopsis* [15], wheat [16, 17] and grape [18], have significantly increased the understanding of the molecular-genetic basis of host resistance in the context of PM infection. Xin et al. [16] reported that a wide range of pathways were initiated following PM inoculation such as cell wall fortification, flavonoid biosynthesis, and metabolic processes. More recently, RNA-seq was used to identify genes whose expression correlated with PM resistance in a panel of two wild and five cultivated central Asian grape accessions. This revealed that resistant accessions were characterized by an early up-regulation of 13 genes, most encoding putative defense functions [18]. Little work has been done, however, to study the transcriptomic responses of cucumber to PM on a genomic scale.

Segment substitution lines (SSLs) are very powerful resources that can be used for genetic dissection of QTLs, functional genomics studies and molecular breeding, and they have been applied by plant biologists too explore the genetics of many crops [19, 20]. With high level genetic background uniformity except at the substituted segment(s), all the phenotypic variations between SSLs and the recurrent parent are ostensibly caused by the substituted segment(s). We thus developed a series of SSLs with different PM resistances introgressed from PM tolerant donor Jin5-508 in the genetic background of PM susceptible D8 by marker-assisted selection with sequence characterized amplified regions (SCAR) markers and simple sequence repeats (SSRs) [21]. After eleven generations maker-aided backcrossing with D8, one such SSL, namely SSL508-28, exhibited high resistance to PM. Transcriptome analysis of SSL508-28 during PM infection will illuminate genes and gene pathways related to PM resistance, and help identify candidate PM-resitance genes that can be used to more efficiently produce PM-resistance cucumber cultivars. In this study, whole genome resequencing of SSL508-28 and its donor parents Jin5-508 and D8 was performed to identify the exact boundaries of the breakpoints for the introgression of PM resistance. RNA-seq of the leaves of SSL508-28 and D8 after 48 h of PM inoculation, along with uninfected control leaves from each, was used to identify differentially expressed genes (DEGs) and uncover the candidate genes that may be underlying cucumber PM resistance.

## Results

### Performance of powdery mildew resistance

**Fig. 1:**
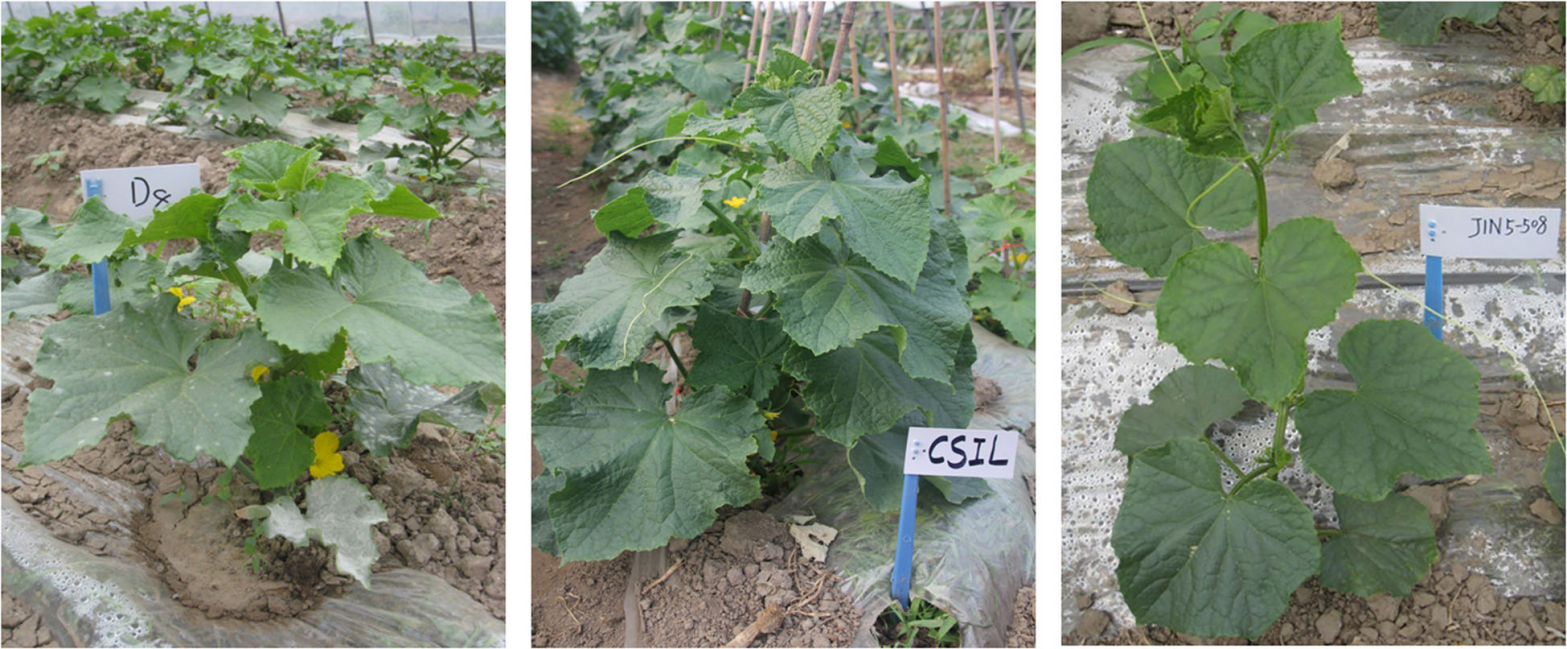
Morphology and powdery mildew (PM) natural infestation responses of D8 (PM susceptible recurrent parent, left), Jin5-508 (PM resistant donor, right), and the derived chromosome segment introgression line (CSIL), named segment substitution line SSL0.7 (PM resistant, middle), after SSR markers identification

**Fig. 2:**
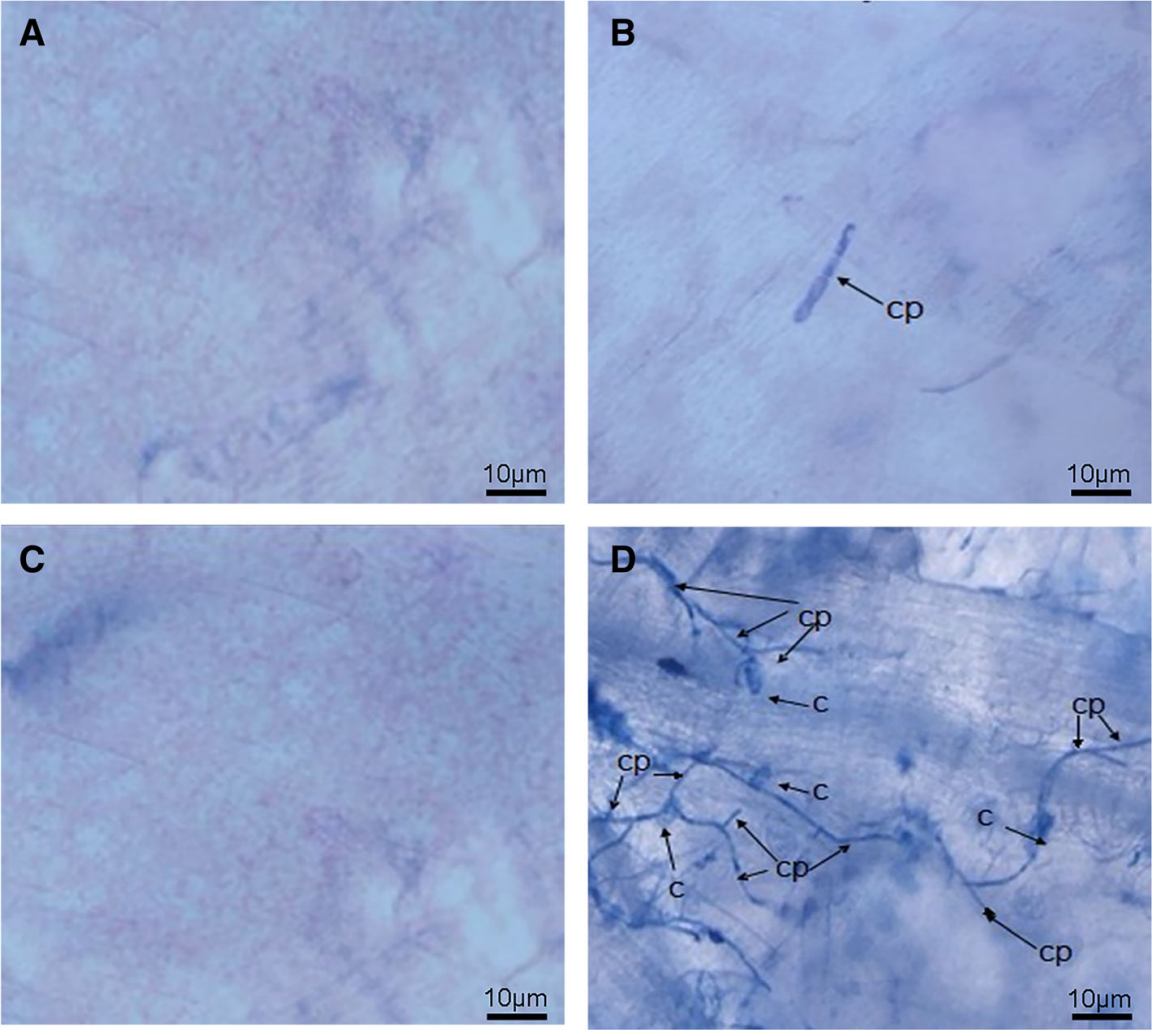
Microscopic observations of uninoculated leaves of SSL508-28 (**a**), 48 h of PM inoculated leaves of SSL508-28 (**b**), uninoculated leaves of D8 (**c**) and 48 h of PM inoculated leaves of D8 (**d**). The arrow indicates the developing PM pathogen. The figure shows representative leaf images from three biological replicates. c, conidium; cp, conidium peduncles

There were no visible differences between SSL508-28 and the recurrent parent D8 in terms of plant morphology and plant height, except that SSL508-28 was highly resistant to PM (Fig. 1). The disease index (DI) for D8 after 15 days of PM pathogen inoculation was 30.6, 37.7 and 36.7 in 2013, 2014 and 2015, respectively. Under the same environmental conditions, SSL508-28 showed consistent higher resistance to PM with DIs of 1.3, 0.7 and 1.3 over the 3 years. To better describe the resistant phenotype difference of SSL508-28 and D8, fungal growth was cytological assessed 48 h after inoculation with PM pathogen. No conidium was detected in either of the control lines (Fig. 2a, c). Microscopic observation showed no hyphal network and the appearance of only one conidium peduncles (cp) on the leaves of SSL508-28, whereas a dense hyphal network with a relatively high number of spore-containing cp formed on the leaves of D8 (Fig. 2b, d). These results suggest that differences in the resistance mechanisms may contribute to the different phenotypes of SSL508-28 and D8.

### Molecular delimitation of substituted segment length in SSL508-28

The introgressed fragment in SSL508-28 was delimited by two SSR markers, SSR15321 and SSR00170, but the exact boundaries of the breakpoints for this introgression were not known because of the limited number, and low density of available SSR markers in cultivated cucumbers. To more precisely estimate the length of the introgressed fragment, whole-genome resequencing of SSL508-28, recurrent parent D8, and donor parent Jin5-508 was performed to identify high-quality single-nucleotide polymorphisms (SNPs) and insertions and deletions (InDels). A total of 108.96 million, 108.89 million and 94.06 million paired-end reads were obtained for SSL508-28, D8, and Jin5-508, respectively. The average sequence depth was 36-fold in SSL508-28, 31-fold in D8, and 21-fold in Jin5-508 (Table 1). A total of 535,270, 553,194 and 161,811 SNPs, as well as 110,457, 120,858 and 30,447 InDels were identified in SSL508-28, D8, and Jin5-508, respectively, by comparing each individually with the cucumber 9930 reference genome assembly. 414,442, and 450,504 SNPs, as well as 70,378 and 81,778 InDels, were identified in SSL508-28, D8, respectively, by comparing each individually with Jin5-508. In contrast, only 18,943 SNPs, and 4,810 InDels were identified between SSL508-28 and D8 (Tables 2 and 3). Between SSL508-28 and D8, a total of 133 SNPs were non-synonymous mutations in 61 genes, and 10 InDels were present in exonic regions of 10 genes. Of these, four genes were found to have both SNPs and InDels in their exonic regions (Additional file 1: Table S1). Approximately 94%, or 63, of these genes were located on chromosome five, which strongly suggests a segment introgression in chromosome five of SSL508-28, with the remaining genetic background being almost identical to D8.

**Table 1:** Summary of whole-genome resequencing data of SSL508-28, D8 and Jin5-508

Mapping Statistics	SSL508-28	D8	Jin5-508
Total reads	108,955,888	108,882,322	94,060,146
Mapped reads	86,565,453	84,895,546	71,081,252
Properly paired	77,587,488	73,060,038	58,956,899
Average depth	36	31	21
Q30 Percentage	94.41%	95.18%	94.57%

**Table 2:** Comparison of single-nucleotide polymorphisms among SSL508-28, D8, Jin5-508 and the cucumber reference genome (9930 V2)

	SSSL508-28	D8	Jin5-508	9930
SSSL508-28	0	18,943	414,442	110,457
D8	18,943	0	450,504	553,194
Jin5-508	414,442	450,504	0	161,811
9930	110,457	553,194	161,811	0

**Table 3:** Comparison of insertions and deletions among SSL508-28, D8, Jin5-508 and the cucumber reference genome (9930)

	SSSL508-28	D8	Jin5-508	9930
SSSL508-28	0	4,810	70,378	535,270
D8	4,810	0	81,778	120,858
Jin5-508	70,378	81,778	0	30,447
9930	535,270	120,858	30,447	0

**Fig. 3:**
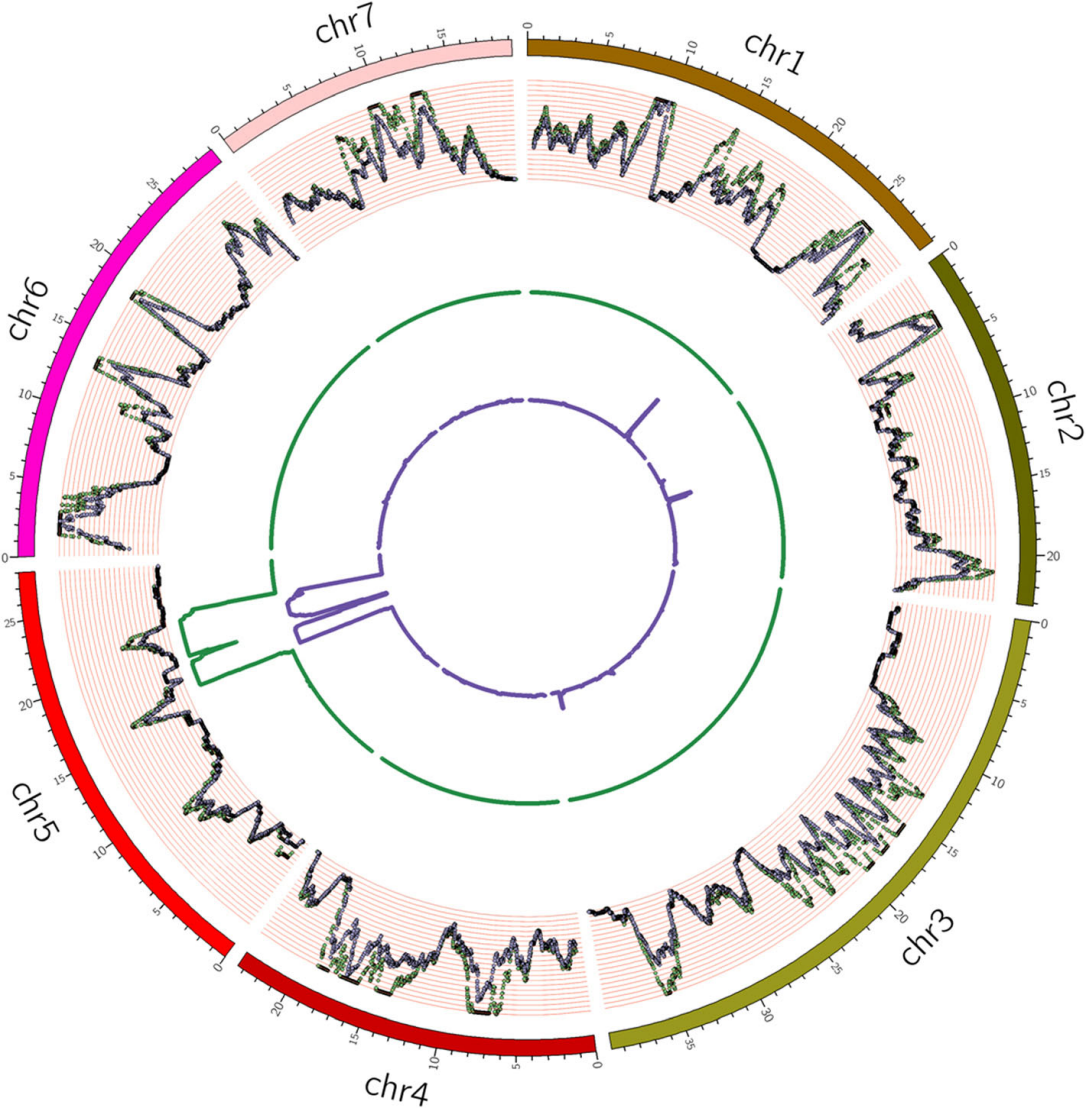
Circos plot representing the location of the introgressed segment. The outer ring represents the seven cucumber chromosomes. The scatter plot inside this ring shows the distribution of homozygous SNPs and InDels between Jin5-508 and D8 across the cucumber chromosomes, SNP: *green*; InDel: *purple*. The *green line* represents the genotype percentage of SNPs in SSL508-28 from the donor parent Jin5-508 along the chromosomes; the inner *blue line* represents the genotype percentage of InDels in SSL508-28 from the donor parent Jin5-508 along the chromosomes

To examine the location of the introgressed segment in SSL508-28 in detail, genotype percentages of SNPs and InDels in SSL508-28 from donor parent Jin5-508 and recurrent parent D8 were calculated along the chromosomes in 100 kb sliding windows (Fig. 3, Additional file 2: Table S2). Screening removed homozygous SNPs and InDels between Jin5-508 and D8 with less than 5-fold read depths. Interestingly, one region on chromosome five was identified as the introgressed segment by examining the genotype percentages of SNPs. The region that we named *Pm5.1* spanned 6.8 Mb (16,676,542 bp to 23,484,079 bp) and contained 3016 SNPs. There were two regions on chromosome five that were identified as introgressed segments by examining the genotype percentages of InDels, one segment spanned 2.0 Mb (16,955,739 bp to 18,982,159 bp) and contained 155 InDels, the other spanned 2.9 Mb (20,456,419 bp to 23,329,026 bp) and contained 215 InDels. The segments identified using the InDels fell within the segment identified using the SNPs, suggesting that the 6.8 Mb segment represents the exact boundaries of the breakpoints for this introgression. The introgressed segment *Pm5.1* contains 856 genes.

### Transcriptome profile based on RNA-seq in SSL508-28 and D8

**Fig. 4:**
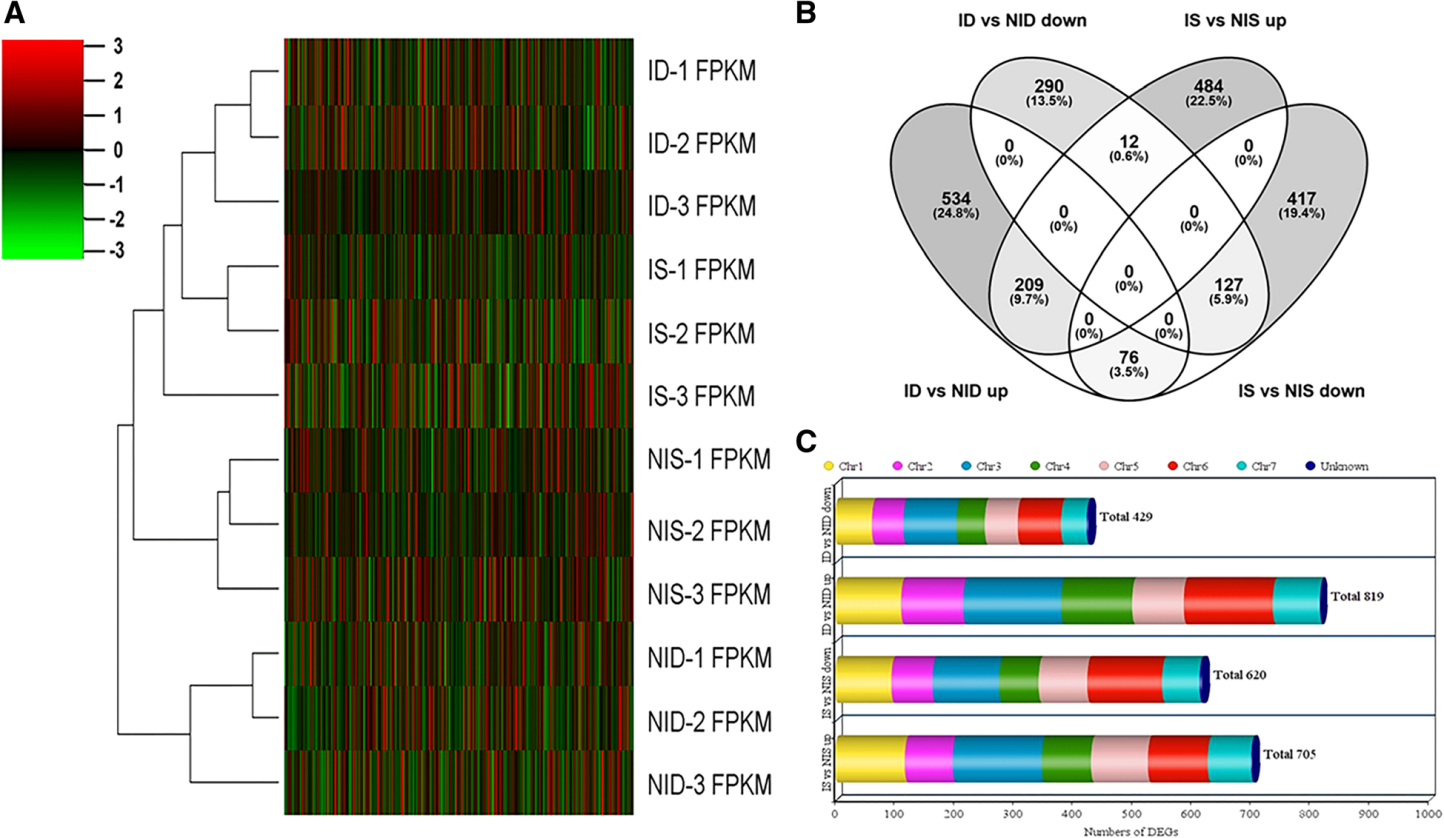
Expression patterns of expressed cucumber genes. **a** Hierarchical cluster of expressed cucumber genes in 12 samples. In the color panels, each vertical line represents a single gene and the color of the line indicates the expression level of the gene relative to the mean center in a specific sample: high expression level in *red*, low expression level in *green*. ID = inoculated D8, NID = non-inoculated control D8, IS = inoculated SSL508-28, NIS = non-inoculated control SSL508-28 and FPKM = fragments per kilobase million. **b** Venn diagram showing the distribution of differentially expressed genes. ‘ID vs NID up’ stands for genes upregulated in D8 48 h after PM inoculation when compared with its un-inoculated control; ‘ID vs NID down’ stands for genes downregulated in D8 48 h after PM inoculation when compared with its un-inoculated control. ‘IS vs NIS up’ stands for genes upregulated in SSL508-28 48 h after PM inoculation when compared with its control; ‘IS vs NIS down’ stands for genes downregulated in SSL508-28 48 h after PM inoculation when compared with its control. **c** Distribution of differentially expressed genes (DEGs) on each chromosome of cucumber

To identify PM responsive genes in SSL508-28 and D8, the transcriptomes of normally watered control leaves and leaves inoculated with PM for 48 h from these two lines were characterized using RNA-seq. A total of 12 standard Illumina cDNA libraries, including three biological replicates for each line at each stage, were prepared for RNA-seq. After removing the adaptor-containing, unknown (where the proportion of undetermined bases was >10%), and low-quality reads, a total of approximately 566 million paired-end reads were obtained, with an average of 47.2 million reads per sample. Approximately 87% (492 million) of these reads could be uniquely mapped to the cucumber reference genome. The assembled transcripts were further filtered based on expression levels of greater than 0 fragments per kilobase million (FPKM). In total, the expression of 22,976 transcripts was detected in the 12 tested samples. The expressed genes from all 12 samples were subjected to a cluster analysis. As shown in Fig. 4a, the two genotypes after 48 h of PM inoculation and in the control were separated from each other. The six control samples clustered into one group, and the inoculated samples clustered into another group, suggesting that most of the expressed genes had similar expression patterns in response to PM infection, even in different genotypes. The inoculated D8 and SSL508-28 clustered into different subgroups, indicating that some gene expression responses to pathogen infection were different.

**Fig. 5:**
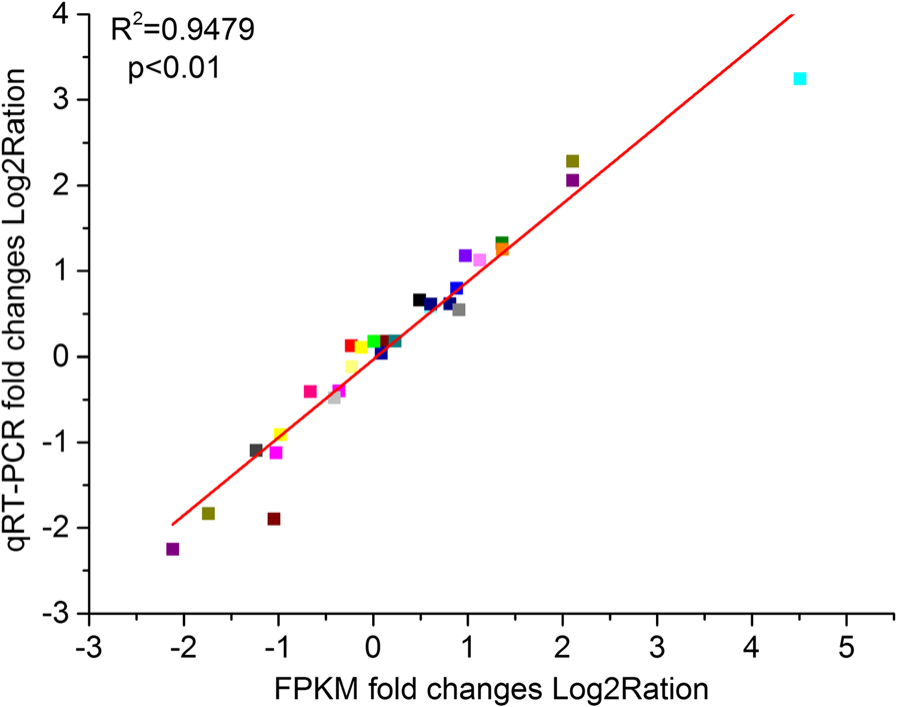
Comparison of transcription levels measured by RNA-sequencing and quantitative real-time reverse transcription-PCR (qRT-PCR) assays. The gene expression values were transformed to the log2 scale. The fragments per kilobase million (FPKM) fold changes log2-values (X-axis) were plotted against the qRT-PCR fold changes log2-values (Y-axis). The cucumber *β-actin* gene (GenBank AB010922) was used as an internal control to normalize the expression data. Each value denotes the mean relative level of expression of three biological replicates

The normalized expression levels in uninfected control and PM inoculated plants were compared to detect DEGs. A total of 1248 DEGs, including 819 upregulated and 429 downregulated genes, were obtained after comparing the PM inoculated D8 (ID) leaves to the non-inoculated D8 (NID) control leaves (Additional file 3: Table S3). In contrast, 1325 DEGs, including 705 upregulated and 620 downregulated genes, were identified after comparing the inoculated SSL508-28 (IS) leaves against the non-inoculated SSL508-28 leaves (NIS) (Additional file 4: Table S4). Seventy six genes were upregulated in ID vs NID, but downregulated in IS vs NIS; 12 genes were downregulated in ID vs NID but upregulated in IS vs NIS (Fig. 4b). The DEGs distributed in all seven cucumber chromosomes (Fig. 4c). To confirm the regulation of the identified DEGs, the expression trends of 15 representative genes were evaluated by quantitative real-time PCR (qPCR) in a separate experiment. The strong correlation (R^2^ = 0.9479, *p* < 0.01) between RNA-seq and quantitative real-time PCR expression values indicated that there was a high level of agreement between the approaches (Fig. 5).

### Functional Annotation of DEGs

**Fig. 6:**
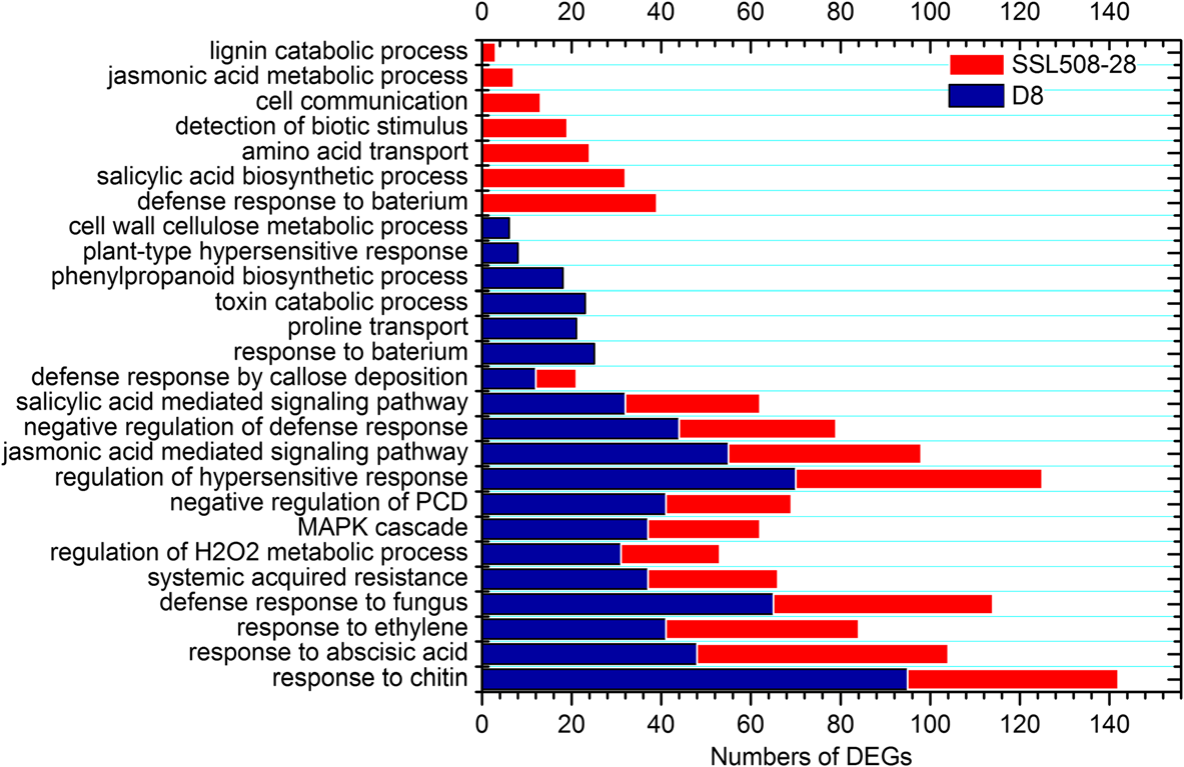
Comparison of selected significant enriched gene ontology terms among differentially expressed genes. The *red bar* stands for the differentially expressed genes in SSL508-28 48 h after PM inoculation when compared with its control; the *blue bar* stands for the differentially expressed genes in D8 48 h after PM inoculation when compared with its control. Additional file 4: Table S4 and Additional file 5: Table S5 has a full list of genes and gene ontology analysis

Of the 1248 DEGs identified in the ID vs NID pair-wise analysis and the 1325 DEGs identified in the IS vs NIS analysis, 1071 and 1160 DEGs, respectively, were assigned one or more gene ontology (GO) terms. In both cases, all GO assignments fell into broad categories for the three major GO functional domains (biological processes, molecular functions, and cellular components). A GO term was considered to be significantly enriched if the false discovery rate was below 0.05. Sixty-two significantly different GO annotations were obtained for the ID vs NID pair with 52 belonging to biological processes, six belonging to molecular functions and four belonging to cellular components (Additional file 5: Table S5). In contrast, 87 significantly different GO terms were obtained for the IS vs NIS pair with 71 belonging to biological processes, nine belonging to molecular functions and seven belonging to cellular components (Additional file 6: Table S6). Enrichment analysis of GO functions revealed that 46 GO terms were found shared between the ID vs NID and IS vs NIS DEGs, such as response to chitin (GO:0010200), response to fungus (GO:0009620), intracellular signal transduction (GO:0035556), respiratory burst involved in defense response (GO:0002679), kinase activity (GO:0016301), and plant-type cell wall (GO:0009505). Thirty-two GO terms were unique to the IS vs NIS pair, including detection of biotic stimulus (GO:0009595), systemic acquired resistance (GO:0009862), salicylic acid biosynthetic process (GO:0009863), phosphatidylinositol phosphorylation (GO:0046854) and defense response to bacterium (GO:0042742). These GO terms are normally regarded as being related to disease resistance (Fig. 6, Additional file 5: Table S5 and Additional file 6: Table S6).

In addition to GO term assignment, Kyoto encyclopedia of genes and genomes pathway mapping, based on the encyclopedia’s orthology terms for assignments, was also carried out to evaluate the disease-resistance related pathway. Twelve significantly enriched pathways, with a false discovery rate of less than 0.05, related to PM inoculation were identified in the IS vs NIS pair-wise analysis (Additional file 7: Table S7), and 17 significant enriched pathways were identified in the ID vs NID pair-wise analysis (Additional file 8: Table S8). Among these significantly enriched pathways, seven were common to both pairs, including Plant-pathogen interaction (ko04626), Phenylalanine metabolism (ko00360) and Phenylpropanoid biosynthesis (ko00940). Antigen processing and presentation (ko04612) and nucleotide-binding oligomerization domain-like receptor signaling pathway (ko04621), normally regarded as disease-resistant related events, are only found in IS vs NIS pair-wise analysis. These findings suggested a role for these genes in regulating PM resistance mediated by *Pm5.1*.

### DEGs in the 6.8-Mb introgressed segment

The expression patterns of DEGs within the introgressed segment *Pm5.1* could be used to prioritize candidate causal genes as they often affect phenotypic variation through transcriptional regulation. A total of 44 DEGs within the 6.8-Mb introgressed segment, including 30 upregulated and 14 downregulated, were obtained after comparing the PM inoculated D8 leaves to the non-inoculated D8 control leaves. In contrast, 64 DEGs, including 36 upregulated and 28 downregulated genes, were identified after comparing the inoculated SSL508-28 leaves against the non-inoculated SSL508-28 leaves. Shared between two genotypes were eight DEGs (*Csa5G512930*, *Csa5G544060*, *Csa5G551250*, *Csa5GS69870*, *Csa5G592800*, *Csa5G605140*, *Csa5G606540* and *Csa5G606550*) with significantly increased expression and five DEGs (*Csa5G484630*, *Csa5G547610*, *Csa5G548130*, *Csa5G602220* and *Csa5G603940*) with significantly decreased expression. A few genes had opposite regulation, increased expression in one genotype and decreased in another genotype, namely, three DEGs (*Csa5G571580*, *Csa5G576580* and *Csa5G608360*) with increased expressions in SSL508-28 and decreased in D8, and four DEGs (*Csa5G471600*, *Csa5G524780*, *Csa5G591770* and *Csa5G600370*) with decreased expression in SSL508-28 and increased expression in D8. DEGs that fell into the defense response (*Csa5G512930*), regulation of reactive oxygen species (*Csa5G569350*), systemic acquired resistance (*Csa5G600370* and *Csa5G600380*), regulation of plant-type hypersensitive response (*Csa5G 604040*), abscisic acid-activated signaling pathway (*Csa5G606540* and *Csa5G606730*), and innate immune response (*Csa5G606310*) were considered as candidate genes associated with PM resistance in cucumber (Table 4). No DEG within the segment contains non-synonymous SNP or InDel in its exonic region.

**Table 4:** List of eight candidate genes in the 6.8-Mb introgressed segment

#	Gene ID	ID vs NID	FDR	IS vs NIS	FDR	Functions
1	Csa5G600370	0.49	3.97E-08	2.58	0.01	Receptor protein kinases
2	Csa5G600380	1.21	0.35	0.43	7.55E-09	Receptor protein kinases
3	Csa5G569350	1.19	0.66	3.42	2.46E-06	GRAS transcripton factor
4	Csa5G606310	3.69	1.85E-20	1.39	2.69E-03	NAC transcripton factors
5	Csa5G604040	2.22	0.16	2.02	0.03	P-type ATPase
6	Csa5G606540	9.35	3.55E-09	2.46	6.35E-06	Remorin family proteins
7	Csa5G606730	2.29	1.38E-06	8.63	0.31	Remorin family proteins
8	Csa5G512930	9.86	2.12E-137	5.10	9.00E-217	70 kDa heat shock protein

*ID* inoculated D8, *NID* non-inoculated control D8, *IS* inoculated SSL508-28, *NIS* non-inoculated control SSL508-28. ‘ID vs NID’ stands for genes fold changes of D8 after 48 h of PM inoculation when compared with its un-inoculated control. ‘IS vs NIS’ stands for genes fold changes of SSL508-28 after 48 h of PM inoculation when compared with its un-inoculated control

### DEGs in the whole genome related to protein kinases and mildew Locus O (MLO) in SSL508-28 and D8 respond to PM infection

Protein kinases play key roles in the early stage of signal recognition and the subsequent activation of plant defense mechanisms during pathogen infection [22]. PM infection affected the expression of protein kinases in both cucumber genotypes. 85 DEGs encoding protein kinases were identified in the sensitive genotype D8 in response to PM infection; 68 were up-regulated and 18 were down-regulated (Additional file 9: Table S9). In tolerant genotype SSL508-28, exposed to the same condition, 72 DEGs were found of which 41 were up-regulated (Additional file 9: Table S9). Four genes, *Csa5G600370*, *Csa6G350360*, *Csa6G515500* and *Csa7G373530* showed a peculiar behavior; they were up-regulated in the sensitive genotype D8 and dramatically down-regulated in the tolerant genotype SSL508-28. *Cs5G600370* and *Csa6G515500* encode cysteine-rich receptor-like protein kinases (CRKs); *Csa6G350360* encodes wall-associated receptor protein kinase (WRK); *Csa7G373530* encodes inactive leucine-rich repeat receptor-like protein kinase (LRR-RLPK).

*MLO* is a plant-specific gene family, which is known to response to biotic stresses in various plant species. *MLO* genes have been found to be involved in PM susceptibility, such as wheat [23], tomato [24], Arabidopsis [25], pepper [26] and tobacco [27]. For example, presence of the barley *MLO* is an absolute requirement for successful penetration of the host cell wall by the corresponding compatible PM [28]. Two DEGs encoding MLO proteins were differentially expressed in tolerant genotype SSL508-28: one gene (*Csa1G085890*) was up-regulated, the other one (*Csa5G623470*) was down-regulated. In the sensitive genotype D8 two *MLO* genes (*Csa6G078520* and *Csa6G509690*) were found and both of them were up-regulated.

### Differential expression of transcript factors (TFs) in the whole genome of SSL508-28 and D8

**Fig. 7:**
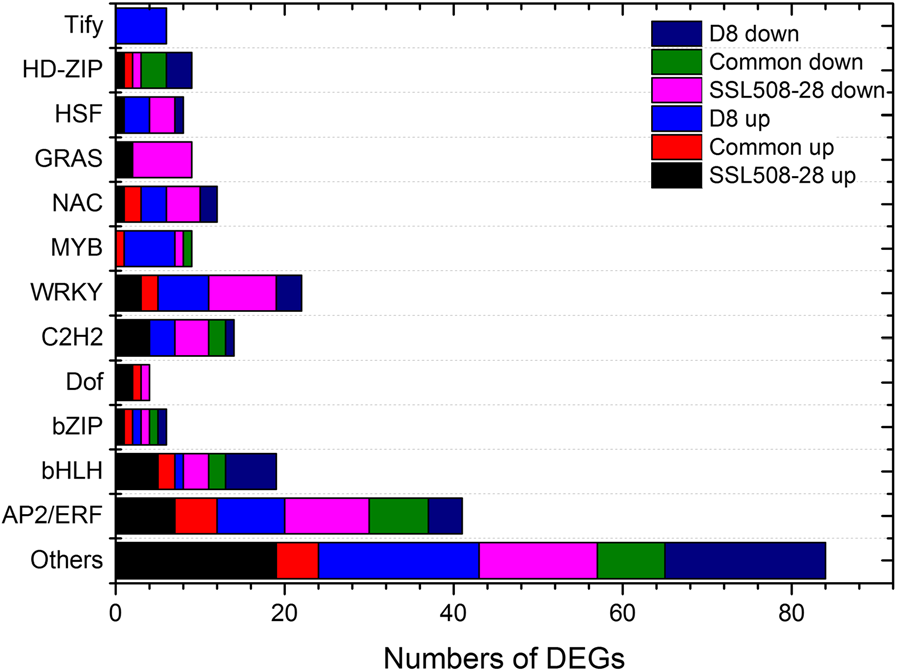
Graphical representation of *Pm5.1*-regulated transcription factors based on their assigned protein families. ‘Common’ represent common regulated DEGs. ‘Up’ and ‘Down’ represent up-regulation and down-regulation in this analysis. Others transcription factors families include BBR-BPC, CSD, NF-YB, ARF, BES1, YABBY, CAMTA, HB-other, Jumonji, Pseudo ARR-B, SNF2, SWI/SNF-BAF60b, zf-HD, HMG, TUB, HB-BELL, RWP-RK, TAZ, DBP, FAR1, GARP-ARR-B, GeBP, GRF, PLATZ, SBP. Detailed information of these families can be seen form plant transcription factor database V3.0 (http://planttfdb.cbi.pku.edu.cn/)

TFs are DNA-binding proteins that control the target gene transcription, and as such play important roles in multiple cellular processes including development, cell cycle regulation, and response to pathogen attacks [29, 30]. 233 TFs were classified based on their assigned protein families, which accounted for nearly ten percent of the DEGs analyzed in present study. Of these, only 43 TFs (20 upregulated and 23 down-regulated) were common regulated in SSL508-28 and D8, whereas 95 TFs (44 upregulated and 51 down-regulated) were regulated in SSL508-28 only, and 86 TFs (49 upregulated and 37 down-regulated) were regulated in D8 only. Remarkly, AP2/ERFs (41), WRKY (22), bHLH (19) were the most frequently identified TFs (Fig. 7). Some TFs, such as HSF, HD-ZIP, bHLH, C_2_H_2_, GRAS, and Tify families that have not previously been associated with diseases resistance, were also significantly induced or repressed. In particular, nine GRAS TFs were found to be dramatically regulated in the tolerant genotype SSL508-28 only, and six Tify TFs were up-regulated in the sensitive genotype D8 only. In this study the WRKY TF *Csa6M486960.1* was found to be upregulated in SSL508-28 but down-regulated in D8. Numerous studies have indicated that WRKY TFs play complicated roles in plant defense signaling. For example, stripe mosaic virus-induced gene silencing of barley *HvWRKY10*, *HvWRKY19*, and *HvWRKY28* compromised resistance-gene-mediated defense to PM [31]. Constitutive over-expression of the *TaWRKY45* in transgenic wheat conferred enhanced resistance against *Blumeria graminis* (a causal fungus for PM) [32]. These observations implicated the important role of TFs in response to PM infection.

## Discussions

Comparative transcriptomic analysis is useful for exploring genes under selection and elucidating the role of various biological pathways and mechanisms for imparting disease tolerance [33]. In the current study, we used a SSL to investigate PM resistance in cucumber. SSL508-28 possessed a segment from the PM resistant donor (Jin5-508), allowing for more precise detection of the PM responsive genes [34]. In PM inoculation tests conducted over 3 years, the resistance of SSL508-28 was found to be highly consistent and stable, suggesting that the introgressed fragment truly carried a PM resistant locus. The introgressed segment, *Pm5.1*, in SSL508-28 was delimited by two previously identified polymorphic SSR markers: SSR15321 and SSR00170 [21]. The exact boundaries of the breakpoints for this introgression and whether there were other minor substituted segments was not, however, known because of the narrow genetic base of cultivated cucumbers and the low density dense of available polymorphic SSR markers in the genome [35, 36]. Based on deep-coverage whole genome resequencing of SSL508, the donor parent Jin5-508, and the recurrent parent D8, we calculated the genotype ratios of SNPs and InDels in SSL508 from the donor and recurrent parents along the chromosomes in 100 kb sliding windows (Fig. 3; Additional file 2: Table S2). The boundaries of the two introgressed segments calculated using the InDels (totally ~4.9 Mb) was shorter than the fragment identified using the SNPs (~6.8 Mb). This may because of the lower frequency of InDels, relative to SNPs, in the cucumber genome. These results are broadly consistent with our previous work, thus confirming that the phenotypic variation in PM resistance between SSL508-28 and D8 was triggered by introgression of the ~6.8 Mb segment rather than by some unknown stochastic factors during the breeding process. The same method may also be useful for the identification of candidate genes in substituted segment boundaries in other studies where the substitute segment is the only potential source of phenotypic variation.

The location of *Pm5.1* on chromosome five in this study was consistent with the major locus for PM resistance identified in the previous studies [6, 12, 13, 37], despite these studies using different populations, types of molecular markers, molecular mapping strategies and phenotyping statistical methods. Therefore, we are reasonably confident that the location of *Pm5.1* identified by SSR and resequencing in our study is correct. Nie et al. [13] cloned a recessive inherited candidate gene for the major QTL on cucumber chromosome five and found that the resistance was caused by the insertion of a 1449-DNA fragment into an *MLO-like* gene (*Csa5G623470*) in the PM resistant line S1003. This MLO-like gene is, however, located in an ~170 kb region between markers UW065021 and UW065094, that is not in the interval of the 6.8 Mb segment. Most importantly, unlike the enhanced expression of *Csa5G623470* in S1003 upon PM inoculation [13], its expression was significantly down-regulated in SSL508-28 (~0.37-fold, Additional file 4: Table S4) after inoculation, indicating the PM defense mechanism in SSL508-28 is different from that reported by Nie et al. [13]. Microscopic observation showed a relatively high numbers of spore-containing cp on the leaves of D8, while the disease symptoms of SSL508-28 were much weaker than those of D8, with only one cp observed 48 h after PM inoculation (Fig. 2). This indicates that some of the genes differentially expressed between SSL508-28 and D8 after 48 h of PM infection might be the causal gene(s) underling *Pm5.1.*

Although it is still hard to determine which DEG is the truly causal genetic factor governing *Pm5.1*, whole-transcriptome sequencing approaches have already provided a narrow pool of candidates for our further investigation. GO annotations revealed that eight DEGs might be the candidates for *Pm5.1* locus from the 856 genes in the 6.8 Mb *Pm5.1* substituted segment (Table 4). Of particular interest within the 9 DEGs were two tandemly arrayed receptor protein kinases (RPKs, *Csa5G600370* and *Csa5G600380*) that fell into GO term ‘systemic acquired resistance’, a GRAS TF (*Csa5G569350*) that fell in to GO term ‘regulation of reactive oxygen species’ and a NAC TF (*Csa5G606310*) that fell into GO term ‘innate immune response’. During the interaction with microorganisms, RPKs active innate immune responses to pathogen attack in plants [38]. In our RNA-seq data, *Csa5G600370* was up-regulated in SSL508-28 (2.57-fold), whereas down-regulated in D8 (0.49-fold), suggesting that *Csa5G600370* might function as a positive regulator of PM resistance in cucumber. Unlike *Csa5G600370*, the other RPK *Csa5G600380* was down-regulated in SSL508-28 (0.43-fold), suggesting distinct functions for these two genes in PM responses. Similar expression pattern divergences of RPKs were also observed in *Arabidopsis* leaves during downy mildew pathogen infection [39] and in wheat during stripe rust fungus inoculation [40]. The GRAS family TF was upregulated ~3.42-fold in SSL508-28. Suppression of *GRAS6* expression impaired tomato resistance to *P. syringae* pv. *tomato* [41]. In addition, tobacco GRAS homologues were reported to be induced upon treatment with hydrogen peroxide, which is well known for its involvement in plant defense responses [42]. The NAC family TF was upregulated ~3.69-fold in D8. A previous study revealed that NAC genes regulated defence response against pathogens by downstream activation or suppression of pathogenesis-related genes. A mutant allele of NAC gene *ATAF1* was found to compromises penetration resistance in *Arabidopsis* against *Blumeria graminis* f. sp. *hordei* [43]. However, transgenic plants in which *ATAF1* was overexpressed showed enhanced susceptibility to *P. syringae* pv. *tomato* DC3000, *Botrytis cinerea*, and *Alternaria brassicicola*, and the *ATAF1* mutant plants showed no significant resistance against the pathogens tested [44]. The candidate gene list contains a gene encoding P-type ATPase (*Csa5G604040*), which was predicted to associate with plant-type hypersensitive response (HR) by GO annotation. Pathogen recognition in plants leads to inhibition of pathogen growth, which is often accompanied in plants by the HRs, a kind of programmed cell death (PCD) localized at the site of attempted pathogen invasion [45]. The P-type ATPase activities in plant cells are closely related to ion homeostasis, apoplastic pH and membrane integrity [46]. Thus, we inferred that the up-regulation of *Csa5G604040* (~2.02-fold) in SSL508-28 might contribute to the PM resistance by a rapid PCD of the infected leave cells. Targeted gene replacement showed that the rice P-type ATPase-encoding gene, *MgAPT2*, is required for rice blast pathogen infection, and for the rapid induction of host defense responses [47]. Additionally, *Csa5G606540* and *Csa5G606730* were also important candidates, and both ostensibly coding for remorin family proteins. Precise biological roles of this family remain to be investigated, but gene expressions data suggest that these proteins might play important functions during responses to biotic stimuli and might be involved in abscisic acid-activated signaling pathway [48]. Rapid induction of *AtREM1.2*, *AtREM1.4*, and *AtREM6.1* transcripts were observed in *Arabidopsis* upon PM infection, suggesting that possible roles in early stages of plant-microbe interactions. Apart from the genes discussed above, *Csa5G512930* encoding a 70 kDa heat shock protein (HSP) might also be a candidate gene. HSP play roles in both compatibility and resistance processes [43], but specific role in pathogen attack is still not clear. Transcriptomic studies on barley leaf revealed that a 70 kDa *HSP* was also significantly induced 12 h after PM inoculation [49].

The comparative transcriptome profiling of SSL508-20 and its recurrent parent D8 here also provided several interesting insights into the molecular defense mechanisms trigged by *Pm5.1*. First, nearly 20% of DEGs encoded for protein kinases and TFs, implying that signal regulation or transduction might play important roles in *Pm5.1*-regulated PM defense. CRKs can regulate immune system as positive or negative regulators in reactive oxygen species/redox signaling and sensing [8]; WRKs allow host cells to perceive their extracellular environment [50]; LRRs contribute to the recognition of diverse pathogen-derived ligands [51]; The mitogen-activated protein kinase (MAPK) may contribute to recognition of invading pathogens by the host leading to rapid activation of a network of MAPK signals transductors, and subsequently resulting in the release of defense compounds, such as phytoalexin [52]. For example, three genes encoding *MAPK*s (*Csa6G006730*, *Csa2G000340* and *Csa1G479630*) were up-regulated in SSL508-28 (Additional file 4: Table S4), whereas only one *MAPK* (*Csa4G082320*) was upregulated in D8 (Additional file 3: Table S3). These CRK, WRK, LRRs, MAPK and TFs signaling cascades occur rapidly after the recognition of effectors by corresponding R proteins in the host and are followed by downstream activation of the defense system. The differential expression of these genes between the SSL508-28 and D8 might, therefore, lead to different reactions to the PM disease. Second, the resistance mediated by *Pm5.1* might be closely related to cell wall modifications. As the first barrier against pathogen attack, the plant cell wall reacts to localized stress by directly apposing substances onto the inner surface. It has been suggested that cell wall modifications elicited by invading pathogens may represent a disease resistance mechanism [16]. Syntaxin is involved in cell wall fortification and can greatly enhance PM resistance in *Nicotiana benthamiana* [53] Our analysis showed that *Csa2G058690* encoding the syntaxin protein was specifically upregulated (~5.26-fold) in SSL508-28 after PM inoculation (Additional file 4: Table S4). Conversely, several genes encoding cellulase and pectinesterase, such as *Csa3G646640*, *Csa7G343850* and *Csa7G405820*, which facilitate plant cell wall breakdown, were upregulated in D8 to a greater extent than in SSL508-28. We, therefore, propose that increased expression of positive cell wall-related genes might reinforce cucumber resistance to PM, and that negative proteins would play an opposing role. Third, the PM resistance mediated by *Pm5.1* might also relate to salicylic acid (SA) signaling pathway. It is believed that plant resistance to biotrophic pathogenesis, including PM, is controlled largely by a type of induced resistance called ‘systemic acquired resistance’ that requires the activation of the SA signaling pathway [54, 55]. In Arabidopsis, systemic acquired resistance through the SA pathway and expression of defense genes have been shown to occur in the plants infected with PM. Mutation of the *SA induction-deficient2-1* (*SID2-1*) gene yields mutants that fail to accumulate SA and show increased susceptibility to the PM pathogen [56]. Interestingly, eight DEGs enriched in SA biosynthesis in GO: 0009697 were specially regulated in SSL508-28 (Additional file 6: Table S6). Therefore, we propose that the biosynthesis of SA in the early stages of PM infection might also contribute to host defense against the pathogen on SSL508-28 leaves.

## Conclusion

With this work we initiated a next generation sequencing based approach to characterize the exact boundaries of the breakpoints for the cucumber PM resistant introgressed segment *Pm5.1*. The comparative RNA-seq based transcriptomes allow us identify eight potential candidate genes that underlie the *Pm5.1* and a complex regulatory network for *Pm5.1* and mediated PM defense mechanisms which included several signal regulators or transducers, cell wall modifications and salicylic acid signaling pathway.

## Methods

### Plant materials

The PM-resistant segment substitution line SSL508-28 was derived from marker-assisted backcrossing [21]. In brief, an indeterminate North China type cucumber inbred line Jin5-508 with PM resistance) was crossed with a semi dwarf American type cucumber inbred line D8. From 2004–2009, the F_1_ progeny of Jin5-508 × D8 were backcrossed with D8 for 11 generations (two generations per year). Starting from BC_2_, at each generation, a SCAR marker linked with PM resistance in Jin5-508 was used to select plants for backcrossing with D8. At BC_11_, 17 individuals were self-pollinated to generate 17 families with a total of 449 plants. These chromosome segment introgression lines (CSILs) were near isogenic at the PM resistant loci as compared with the D8. SSR analysis of these CSILs identified a SSL, SSL508-28, which carried a single chromosome fragment from Jin5-508 and was highly resistant to PM. SSL508-28 was delimited by two SSR markers SSR15321 and SSR00170 in cucumber chromosome 5 with genetic distance of 25.3 cM on the genetic map developed by Ren et al. [57].

### Powdery mildew inoculation and screening

PM resistance of SSSL508-28, Jin5-508 and D8 and was repeatedly evaluated in three years (spring 2013 and 2014, and fall 2015) in the greenhouses of Yangzhou University (Yangzhou, China). PM conidia were collected from naturally-infected D8 plants in the greenhouse. A spore suspension at 10^6^ spores per ml was made by soaking heavily infected leaves in tap water containing 0.01% Tween-20. Inoculation was performed at the 3^rd^ true-leaf stage by spraying the spore suspension evenly on the surface of the seedlings according to Morishita et al. [58]. After inoculation, the plants were maintained in a controlled growth chamber at 25 °C day/20 °C night with a 16-/8-h (light/dark) photoperiod. The percentage of infected area of each leaf of each sample plant was recorded fifteen days after inoculation using a scale of 0–5 (0 = no symptom; 1 = infection area <30%; 2 = 30–60%; 3 = >60–80%; 4 = >80%; and 5 = leaf senesced). DI was calculated as following: DI = ∑(Disease scale × number of leaves of that specific scale)/(number of leaves inoculated × the highest disease grade) × 100.

### Microscopic examination of PM pathogens infection

For better visual observation the difference between SSL508-28 and D8, leaf tissues were cut into small pieces (0.5–1 cm), fixed and decolorized in 95% ethanol for 2 h, then stained with typan blue (Bio-Rad, California, USA) for 15 min and rinsed with ddH_2_O. For microscopic observations, leaf segments were stored in 50% glycerin and examined under a microscope Olympus BX-43 (Olympus Corporation, Tokyo, Japan).

### SNP and InDel identification

The genomes of the two parental lines, Jin5-508 and D8, and SSL508-28 were re-sequenced with the Illumina HiSeq 2500 platform (Illumina, USA). Genomic DNA from healthy leaves of SSL508-28, Jin5-508 and D8 was extracted by PureLink™ quick gel extraction kit (invitrogen, Germany) and its quality and quantity were evaluated using a Bioanalyzer 2100 (Agilent Technologies, USA). 5 μg genomic DNAs were then used for preparation of paired-end sequencing libraries with insert sizes of ~200 to 500 bp following manufacturer’s protocols. The filtered clean reads were mapped to the cucumber 9930 draft genome assembly version 2 (http://www.icugi.org/cgi-bin/ICuGI/index.cgi) using the GATK software package [59] for SNPs and InDels calling. Synonymous (not alter amino acid sequence) and non-synonymous changes (alter amino acid sequence) were identified with the effect predictor of SnpEff v3.1 h [60]. The distribution of SNPs and InDels on each cucumber chromosomes was visualized using Circos [61].

### RNA extraction, mRNA-seq library construction, and sequencing

Total RNA was extracted using RNeasy Mini Kit (Qiagen, Germany) following the manufacturer’s instructions and checked for a RIN number to inspect RNA integrity by an Agilent Bioanalyzer 2100 (Agilent technologies, USA). All 12 samples had RIN values >7. Qualified total RNA were then purified by RNase-Free DNase Set (Qiagen, Germany) following manufacturer’s instruction. The Illumina TruSeq™ RNA Sample Preparation Kit (Illumina, San Diego, CA, USA) was used for mRNA fragmentation, and first-and second-strand cDNA synthesis. A total of 12 standard Illumina pair-end cDNA libraries, including three biological replicates for each line, were constructed for RNA-seq. The libraries were sequenced as 125-bp paired-end reads using Illumina Hiseq2500 according to the manufacturer’s instructions.

### Mapping reads to the reference genome and quantification of gene expression levels

An index of the cucumber reference genome was built using Bowtie v0.12.8 [62], and high-quality nucleotides (Q > 20) were aligned to the reference genome using TopHat v2.0.9 [63]. Cufflinks v2.1.1 [64] was used to count the read numbers mapped to reference gene. To identify DEGs, we ranked genes according to their size and sequencing coverage normalized fragments per kb of exon per million. The log2 fold changes of gene FPKM between two genotypes were tested statistically to determine whether an individual gene expression was altered significantly or not. A criterion of FDR <0.05 and fold changes >2.0 or <0.5 (>1 or < −1 in log2 ratio value) was used for identifying DEGs.

### qRT-PCR confirmation

To validate the RNA-sequencing results, an independent set of samples with three independent biological replicates was collected at 48 h after PM inoculation and relative controls. Total RNA from each sample was isolated by RNAiso Plus (Takara, China), then dissolved in water-DEPC and kept at a final concentration of 1,000 μg/mL using Biophotometer Plus (Expander, Germany). The cDNA sequences of 15 genes downloaded from the cucumber 9930 reference genome (version 2) were used to design primers by Primer 3 software (http://frodo.wi.mit.edu/; Additional file 10: Table S10). Expression levels of 15 cucumber genes were tested in 25-μL reactions using the RealMasterMix (SYBR Green) kit (Tiagen, China) with the following temperature program: 95 °C for 10 s, followed by 40 cycles of 95 °C for 15 s, and then annealing at 52 °C for 30 s. The relative quantization of gene expressions were calculated and normalized to *Actin*.

## Additional files

Additional file 1: Table S1. Detailed information of genes with non-synonymous mutations between SSL508-28 and D8. (XLS 16 kb)
Additional file 2: Table S2. Genotype percentages of SNPs and InDels in SSL508-28 from donor parent Jin5-508. (XLS 1263 kb)
Additional file 3: Table S3. Differentially expressed genes identified by RNA-seq in D8 after 48 h of powdery mildew inoculation. ID = inoculated D8, NID = non-inoculated control D8, FPKM = fragments per kilobase million. (XLS 628 kb)
Additional file 4: Table S4. Differentially expressed genes identified by RNA-seq in SSL508 after 48 h of powdery mildew inoculation. IS = inoculated SSL508-28, NIS = non-inoculated control SSL508-28, FPKM = fragments per kilobase million. (XLS 521 kb)
Additional file 5: Table S5. GO enrichment analysis of differentially expressed genes in D8 after 48 h of powdery mildew inoculation compared with its control. (XLS 140 kb)
Additional file 6: Table S6. GO enrichment analysis of differentially expressed genes in SSL508-28 after 48 h of powdery mildew inoculation compared with its control. (XLS 179 kb)
Additional file 7: Table S7. Significant Kyoto encyclopedia of genes and genomes pathway of differentially expressed genes in SSL508-28 after 48 h of powdery mildew inoculation compared with its control. (XLS 38 kb)
Additional file 8: Table S8. Significant Kyoto encyclopedia of genes and genomes pathway of differentially expressed genes in D8 after 48 h of powdery mildew inoculation compared with its control. (XLS 48 kb)
Additional file 9: Table S9. Differentially expressed genes encoding protein kinases in SSL508-28 and/or D8 after 48 h of powdery mildew inoculation. (XLS 54 kb)
Additional file 10: Table S10. Detailed primer sequences for qRT-PCR confirmation. (XLS 12 kb)

## Abbreviations

cp: conidium peduncles
CRKs: cysteine-rich receptor-like protein kinases
DEGs: differentially expressed genes
FPKM: fragments per kilobase million
GO: gene ontology
InDels: insertions and deletions
ID: inoculated D8
IS: inoculated SSL508-28
LRR-RLPK: leucine-rich repeat receptor-like protein kinase
NID: non-inoculated D8
NIS: non-inoculated SSL508-28
PM: Powdery mildew
qPCR: quantitative real-time PCR
QTLs: quantitative trait locus
RNA-seq: RNA-sequencing
SCAR: sequence characterized amplified regions
SSL: segment substitution line
SSRs: simple sequence repeats
SNPs: single-nucleotide polymorphisms
TFs: transcript factors
WRK: wall-associated receptor protein kinase
